# A Silicea Case

**Published:** 1887-11

**Authors:** 


### A SILICEA CASE.

Two cats had a fight. One of them was *our* cat. *Our* cat gained the victory, but he received a wound in his left cheek, inflicted by the dirty claws of the other cat. The wound, being thus poisoned by the inoculation of septic matter, speedily degenerated into an ulcer, from which flowed *an ichorous, offensive pus.* So offensive was the odor that we were obliged to exclude his catship from our highly desirable society—greatly to his surprise and displeasure. The ulcer also increased, it is probable, by reason of his constitutional tendency to mange. He had only just recovered from an attack of the latter under Bell.$^{2c}$. For the offensive ulcer we now prescribed Silicea$^{2c}$, two doses. In three days the odor was entirely removed; in a week the ulcer was practically healed, and he was restored to his place in the "bosom of the family."

Of course, the remedy given here was not (?) *scientific!* It was empirical, and possibly the ulcer would have gotten well in any event. We know that to be perfectly "scientific" we should have washed the ulcer with some disinfectant to "kill" the germs that were introduced and propagated from a dirty claw; or, to suit the views of the lady doctors who seceded from the Woman's Hospital, we should perhaps have shut the cat in a room and then have "saturated" the atmosphere of the room with the "vapors" (!) of Platt's Chlorides.

Dr. de Baun's case of "Offensive Feet," similarly, was not a "scientific" prescription. It was "empirical," and the Doctor should never have made the cure—still less have published it. Like ourselves, he does not know any better, so our eclectic friends will have to excuse us both for our ignorance of true "science." W. M. J.

